# Assessment of Electrophoretic Mobility Determination in Nanoparticle Analysis: Two Parallel Techniques Converging in a Distinctive Parameter

**DOI:** 10.1002/elps.202400132

**Published:** 2025-04-29

**Authors:** Carlos Adelantado, Jan Jordens, Stefan Voorspoels, Milica Velimirovic, Kristof Tirez

**Affiliations:** ^1^ Flemish Institute for Technological Research (VITO) Mol Belgium

**Keywords:** capillary electrophoresis, electrophoretic mobility, Laser Doppler velocimetry, nanoparticles

## Abstract

A critical comparison of the main parameters playing a role in measurement of electrophoretic mobility of plastic nanoparticles (NPs) by CE and laser Doppler velocimetry (LDV) techniques in NP suspensions is herein presented, accompanied by a discussion about potential impact on different mobility values observed. Capillary material and dynamic or permanent coating of the inner capillary wall, capillary dimensions, EOF variability, BGE temperature, Joule heating, and presence of species potentially interacting with analyzed NPs are underlined as possible causes of the different performance of the above two techniques. It is of importance to get an insight into the reasons behind experimental conditions and operating features to opt for one technique or the other based on research interests. In the end, it is intended to present a knowledge expansion about two parallel paths that converge in a distinctive parameter of an enormous relevance in CE, effective electrophoretic mobility, not achievable by other techniques, and discuss practical considerations in experimental design.

**Abbreviations**
LDV: laser Doppler velocimetry
NP: nanoparticle
PC: polycarbonate
SCD: surface‐charge density

## 1 Introduction

Electrophoretic mobility is a crucial parameter in determining CE separation of analytes [1]. Apart from CE, there is an alternative, well‐established technique to measure electrophoretic mobility by different principles, but providing this parameter with the same dimension (m^2^ V^−1^ s^−1^). Laser Doppler velocimetry (LDV) determines electrophoretic mobility of particles with applicability to life science, and commercial devices are available to integrate this technique [2]. It employs dynamic laser light scattering in a microtube under electrophoresis conditions to determine particle electrophoretic mobility. The majority of LDV systems use two beams focused and crossed at a point in space forming a small measurement volume with an interference fringe pattern. When a small particle passes through the probe volume, the amplitude of the scattered signal intensity varies due to the fringe pattern. This beat frequency is measured with a photomultiplier tube and filtered [3]. Alternatively, CE offers the possibility of simultaneously measuring electrophoretic mobility of various particle diameters for the same material, a fact that places CE in an advantageous position in comparison to LDV. The former is able to resolve or separate particle mixtures under suitable BGE conditions, whereas the latter can only measure one species at a time; in other words, it provides a single value of electrophoretic mobility, regardless of sample composition. To the best of authors’ knowledge, there is scarce literature on critical comparison among key variables influencing electrophoretic mobility acquisition by both techniques, even if some reports have been described to date on experimental determination of electrophoretic mobility with nanoparticles (NPs) [2, 4, 5, 6]. Moreover, an assessment in the field of plastic NPs of small sizes (tens, hundreds of nm) has not even been conducted to date. Particularly, this journal has recently published interesting articles dealing with electrophoretic behavior of polystyrene (PS) sub‐micron and microparticles by dielectrophoresis [7, 8], together with other research works involving the use of CE for gold NPs [9] and carbon dots [10]. In view of the scope of this journal and the niche found by authors in the field of plastic NPs, it was considered of relevance to compose the proposed manuscript for the interest of the scientific community. The objective of this work is to highlight similarities and differences between both techniques for mobility determination of plastic NPs for sizes below 300 nm, how one technique may complement the other, and vice versa.

## 2 Materials and Methods

### 2.1 Nanoparticles and Chemicals

Non‐functionalized PMMA spheres (40, 100, and 200 nm) were supplied by Lab 261 (Palo Alto, CA, USA) as 1% m/v solid suspension in water. Non‐functionalized PS spheres (30, 60, 90, 200, and 300 nm) were obtained from Distrilab (the Netherlands), as 1% m/v solid suspension in water. Sodium phosphate dibasic (≥99.0%) was obtained from Sigma Aldrich (MO, USA). Sodium dodecyl sulfate (≥99%) was purchased from Fluka (Switzerland). Brij 35 solution (30% m/m in water) was obtained from Merck (Darmstadt, Germany). Ammonium hydroxide solution (25%) was supplied by Sigma Aldrich (Germany). Sodium hydroxide aqueous solution (1 M) was supplied by Agilent Technologies (Santa Clara, CA, USA). Ultra‐pure water (18.2 MΩ cm) was dispensed from a Milli‐Q system (Millipore, Burlington, MA, USA).

### 2.2 Devices and Capillaries

#### 2.2.1 CE Setup

Analyses by CE were carried out in an Agilent Model 7100 (Santa Clara, CA, USA) CE instrument equipped with a UV‐vis spectrophotometric DAD detector. Electropherogram data were treated with OpenLab software (version 3.6) from Agilent Technologies. Bare fused silica capillaries with different lengths (40–70 cm) and id (50–100 µm), od being 363 µm, supplied by Agilent Technologies (USA), were employed for all CE analyses. Every day, prior to experimentation, the capillary was flushed 5 min with 1 M NaOH, followed by water for 5 min and 20 min with the background electrolyte applying a 950‐mbar pressure for these steps. Capillary integrity was verified by applying high voltage during a short period. If a stable intensity current could not be obtained, the capillaries were replaced accordingly. Between runs, capillary was pre‐conditioned with 5 min of water and 5 min of background electrolyte prior to next injection.

#### 2.2.2 LDV Setup

Acquisitions of particle hydrodynamic diameter and electrophoretic mobility were performed on a Zetasizer ZS (Malvern Panalytical, Worcestershire, UK), operating in dynamic light scattering (DLS) and LDV modes, respectively. Measurements were carried out using a 4 mW He‐Ne 633 nm laser module operating at 25°C at an angle of 173° (back scattering) electric field strength for LDV set at 25 V cm^−1^, and data were treated by means of Malvern DTS 7.03 software. Polycarbonate (PC)‐folded cells (DTS1070) supplied by VWR (Belgium) were employed. Prior to first use, PC cells were rinsed with ethanol, followed by water, suspending medium, and the particle suspension of interest. Between different measurements, solely rinsing with water and the particle suspensions was indicated.

### 2.3 Calculations

For both polymeric NP types, migration times were first recorded from CE electropherograms, and effective electrophoretic mobility (*µ*_eff_) values were calculated making use of the following equations:

(1) $$\mu_{\mathrm{eff}}=\mu_{a}\;-\mu_{\mathrm{eof}}$$

(2) $$\mu_{a}=\frac{l\;L}{V\;t}\;$$

(3) $$\mu_{\mathrm{eof}}=\frac{lL}{Vt_{\mathrm{eof}}}$$

**FIGURE 1:**
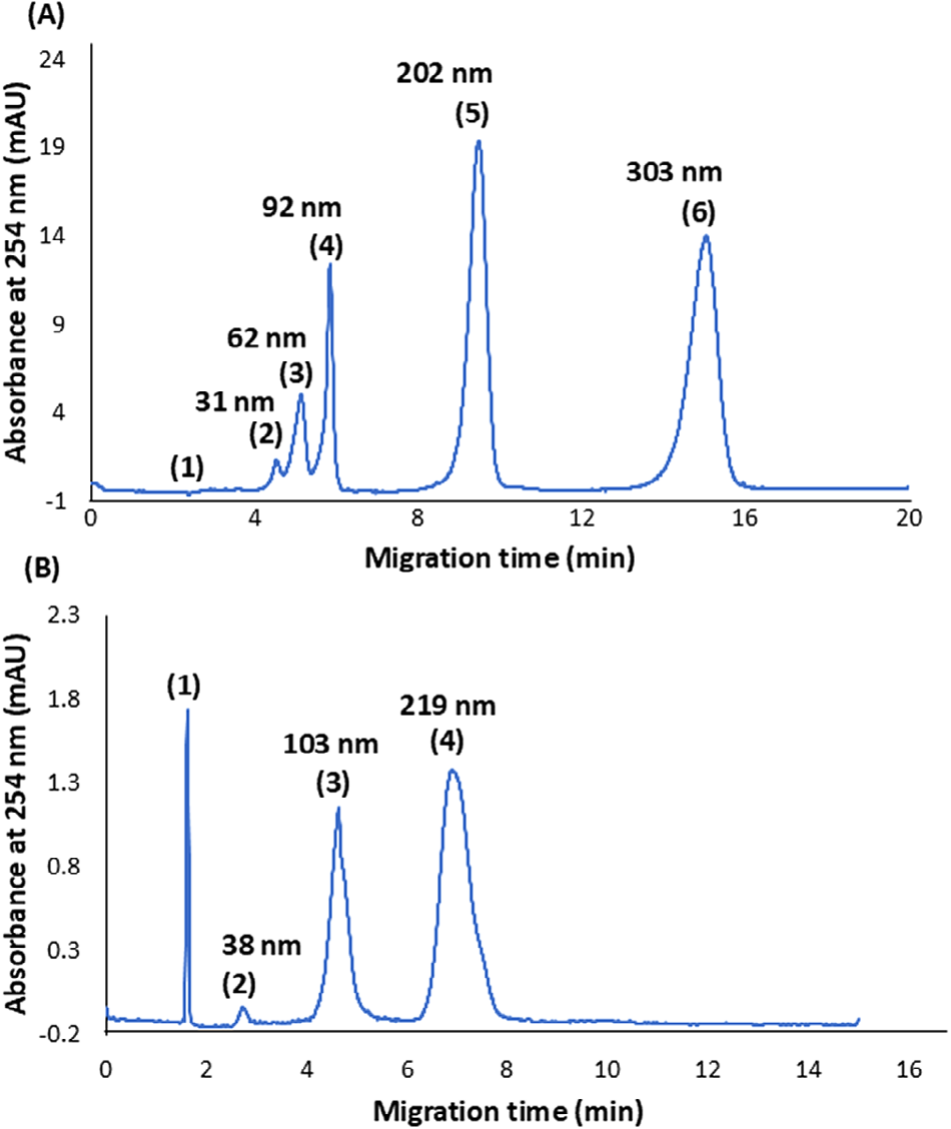
CE separation of (A) PS NPs. Peak identification: (1) EOF marker; (2) 31‐nm PS 6.74 × 10^12^ particles mL^−1^, (3) 62‐nm PS 8.43 × 10^11^ particles mL^−1^, (4) 92‐nm PS 2.50 × 10^11^ particles mL^−1^, (5) 202‐nm PS 2.27 × 10^10^ particles mL^−1^, and (6) 303‐nm PS 6.74 × 10^09^ particles mL^−1^. Main conditions are as follows: bare fused silica capillary (65‐cm length, 56.5 cm effective × 100 µm id x 363 µm od); cassette temperature 25°C; hydrodynamic injection during 5 s at 50 mbar; separation voltage 30 kV; electric current 48 µA; BGE 5 mM sodium phosphate dibasic with 5 mM sodium dodecyl sulfate at pH 8.9. (B) PMMA NPs. Peak identification: (1) EOF marker, (2) 38‐nm PMMA 1.30 × 10^12^ particles mL^−1^; (3) 103‐nm PMMA 1.62 × 10^11^ particles mL^−1^; (4) 219‐nm PMMA 2.02 × 10^10^ particles mL^−1^. Main conditions are as follows: fused silica capillary (50 cm length, 41.5 cm effective × 75 µm id x 363 µm od); cassette temperature 25°C; hydrodynamic injection during 5 s at 50 mbar; separation voltage 28 kV; electric current 43 µA; BGE 2.14 M ammonium hydroxide at pH 11.9.

Here, apparent mobility (*µ*_a_) for each particle diameter was calculated considering effective capillary length (*l*), total capillary length (*L*), separation voltage (*V*), and migration time (*t*). Mobility for EOF (*µ*_eof_) was estimated by the same parameters, with the only exception of migration time for EOF (*t*_eof_). The latter was obtained from the solvent peak, exhibited in the first signal that was assigned in the electropherograms shown in Figure 1.

To calculate the injected volume in CE using hydrodynamic injection, Hagen–Poiseuille equation [11] was applied:

(4) $$V_{c}=\frac{\Delta P\pi d^{4}t}{128\eta L}\;$$

Herein, *V*_c_ is the calculated injection volume, Δ*P* is the pressure difference between the ends of the capillary, *d* is capillary id, *t* is the injection time, *η* is dynamic viscosity of the solution, and *L* is total capillary length (m).

The relationship between the electrokinetic (zeta) potential (ζ) and *µ*_eff_ values is given by Henry equation [1]:

(5) $$\mu_{\mathrm{eff}}=\frac{\varepsilon \zeta }{\eta }\;H\left(\kappa r\right)\;$$

Herein, *ԑ* is the electric permittivity of the solution, obtained as a product of the relative permittivity of the solution (*ԑ*_r_) and the permittivity of vacuum (*ԑ*_0_) in Equation (6)

(6) $$\varepsilon =\varepsilon_{r}\;\;\times \;\varepsilon_{0}$$

ζ is electrokinetic (zeta) potential for the particles, *ŋ* is the dynamic viscosity of the solution, and *H*(*κr*) is Henry function, where *κ* is the inverse Debye length. The Debye length, expressed as 1/*κ*, is the thickness of the electrical double layer around the particle and *r* is particle radius. The function *H* is monotonic, ranging from 2/3 to 1, in the scenario of small particles surrounded by a wide layer of ionic atmosphere. In this study, Smoluchowski approximation for *H* function (1.5) was selected as particles were considered sufficiently large, and ionic atmosphere layer was assumed as narrow.

Particle charge (*q*) was estimated from Equation (7), and surface‐charge density (SCD), defined as *σ*, from Equation (8), as a function of particle charge in a scenario of spherical particles.

(7) $$q=\mu_{\text{eff}}6\pi r$$

(8) $$\sigma =\frac{q}{4\pi r^{2}}=\frac{6\mu_{\text{eff}}\pi r}{4\pi r^{2}}=\frac{3\mu_{\text{eff}}}{2r}$$

Herein, *ŋ* is dynamic viscosity of the solution and *r* is particle radius. It is remarkable to point out that these equations are solely valid for a limiting particle mobility, this being measured at nearly null ionic strength. As a consequence, the usage of these equations with the effective mobilities provided for NPs at the ionic strength given by the BGE implies that the calculation provides only approximate values of *q* and SCD.

## 3 Results and Discussion

### 3.1 Separation of PS and PMMA NPs by CE

The initial hypothesis that gave rise to this research is a recent publication in this group [12], in which PS and PMMA NPs of different diameters have been separated by CE‐DAD. New data for these polymeric NPs have been acquired and are presented next. At first, a mixture of PS NPs of five different diameters (30, 60, 90, 200, and 300 nm) suspended in water, particle concentration ranging from 10^09^ to 10^12^ particles mL^−1^, was separated by CE in a 65 cm bare fused silica capillary (56.5‐cm effective length, 100 µm id, and 363 µm od) with a BGE containing 5 mM sodium phosphate dibasic and 5 mM SDS, pH 8.9. Electric field strength applied during separation was 460 V cm^−1^, provoking an electric current of 48 µA, cassette temperature was set at 25°C, and hydrodynamic injection took place during 5 s at 50‐mbar external pressure. Detection by DAD was performed at different wavelengths, from 200 to 254 nm. A representative electropherogram for separation of PS NPs by their diameter is depicted in Figure 1A.

In an analogous way, a mixture of PMMA NPs of three different diameters (40, 100, and 200 nm) suspended in water, particle concentration ranging from 10^10^ to 10^12^ particles mL^−1^, was separated by CE through a 50‐cm capillary (41.5‐cm effective length, 75 µm id, and 363 µm od) with a BGE containing 2.14 M ammonium hydroxide, pH 11.9. Electric field strength applied during separation was 560 V cm^−1^, provoking an electric current of 43 µA, cassette temperature was kept constant at 25°C, and hydrodynamic injection was held during 5 s at 50‐mbar external pressure. Detection by DAD was conducted at different wavelengths, from 200 to 254 nm. A typical electropherogram for PMMA NPs separation is shown in Figure 1B.

Values for *µ*_eff_ in CE were calculated by Equations ((1), (2), (3)) and then compared with LDV in terms of trend followed by these polymeric NPs from smaller to larger particle diameters. It was found that values for *µ*_eff_ of plastic NPs, namely, PMMA and PS, differed to a certain extent when measured by LDV and CE, even though the dispersing solution consisted of the same BGE ions, ammonium hydroxide, and phosphate for both particle types, respectively. Non‐linear electrophoresis effects were also present in these electropherograms, with implications as a retarding force in NP separation and an increment in migration time, consequently increasing absolute value of *µ*_eff_. Concerning LDV, the device was able to directly acquire *µ*_eff_ values for each particle diameter. Separate measurements for the individual particle diameters were carried out, with the respective BGE conditions for PS and PMMA. Values for *µ*_eff_ are shown in Table 1 for different diameters of PS and PMMA nanoparticles under selected CE and LDV operating conditions.

**TABLE 1:** Values for *µ*_eff_ of PS and PMMA obtained by CE and LDV under the selected electric field strength and BGE conditions for both techniques.

Particle type	Diameter by supplier (nm)	Hydrodynamic diameter (nm) ± SD (*n* = 5) in water (DLS)	*µ*_eff_ by CE (*n* = 5) (10^−9^ m^2^ V^−1^ s^−1^)	*µ*_eff_ by LDV (*n* = 5) (10^−9^ m^2^ V^−1^ s^−1^)
PS	31 ± 3, *k* = 2^a)^	32.8 ± 0.2	−24.1 ± 1.0^b)^	−36.8 ± 8.9^c)^
PS	62 ± 3, *k* = 2^a)^	68.6 ± 0.9	−29.4 ± 1.2^b)^	−37.6 ± 2.9^c)^
PS	92 ± 3, *k* = 2^a)^	99.6 ± 0.5	−34.2 ± 1.1^b)^	−49.5 ± 2.0^c)^
PS	202 ± 4, *k* = 2^a)^	212.4 ± 0.9	−48.1 ± 1.4^b)^	−66.0 ± 2.9^c)^
PS	303 ± 6, *k* = 2^a)^	325.6 ± 3.2	−55.4 ± 0.8^b)^	−68.7 ± 2.7^c)^
PMMA	38, PDI = 0.092^d)^	37.3 ± 0.7	−39.3 ± 0.3^e)^	−40.5 ± 9.0^f)^
PMMA	103, PDI = 0.036^d)^	125.2 ± 0.7	−55.3 ± 0.1^e)^	−46.3 ± 5.1^f)^
PMMA	219, PDI = 0.006^d)^	207.3 ± 0.5	−64.0 ± 1.7^e)^	−59.3 ± 3.9^f)^

^a)^ Measurement by transmission electron microscopy (*k* = coverage factor for 95% expanded uncertainty intervals).

^b)^ BGE containing 5 mM phosphate + 5 mM SDS (pH 8.9) and measurements carried out at *E* = 460 V cm^−1^.

^c)^ BGE containing 5 mM phosphate + 5 mM SDS (pH 8.9) and measurements carried out at *E* = 25 V cm^−1^.

^d)^ Measurement by DLS (PDI = polydispersity index).

^e)^ BGE containing 2.14 M NH_4_OH (pH 11.9) and measurements carried out at *E* = 560 V cm^−1^.

^f)^ BGE containing 2.14 M NH_4_OH (pH 11.9) and measurements carried out at *E* = 25 V cm^−1^.

### 3.2 Discrepancies Between CE and LDV Data for PS and PMMA NPs Electrophoretic Mobility

According to mobility data values acquired for every single NP item, it was necessary to address the impact of Joule heating, as a high electric field strength may be preventing devices from measuring at 25°C. The thermostating system may contribute to minimize the effect of Joule heating, even though both CE and LDV instrument manufacturers claim temperature control effectiveness without validation.

**FIGURE 2:**
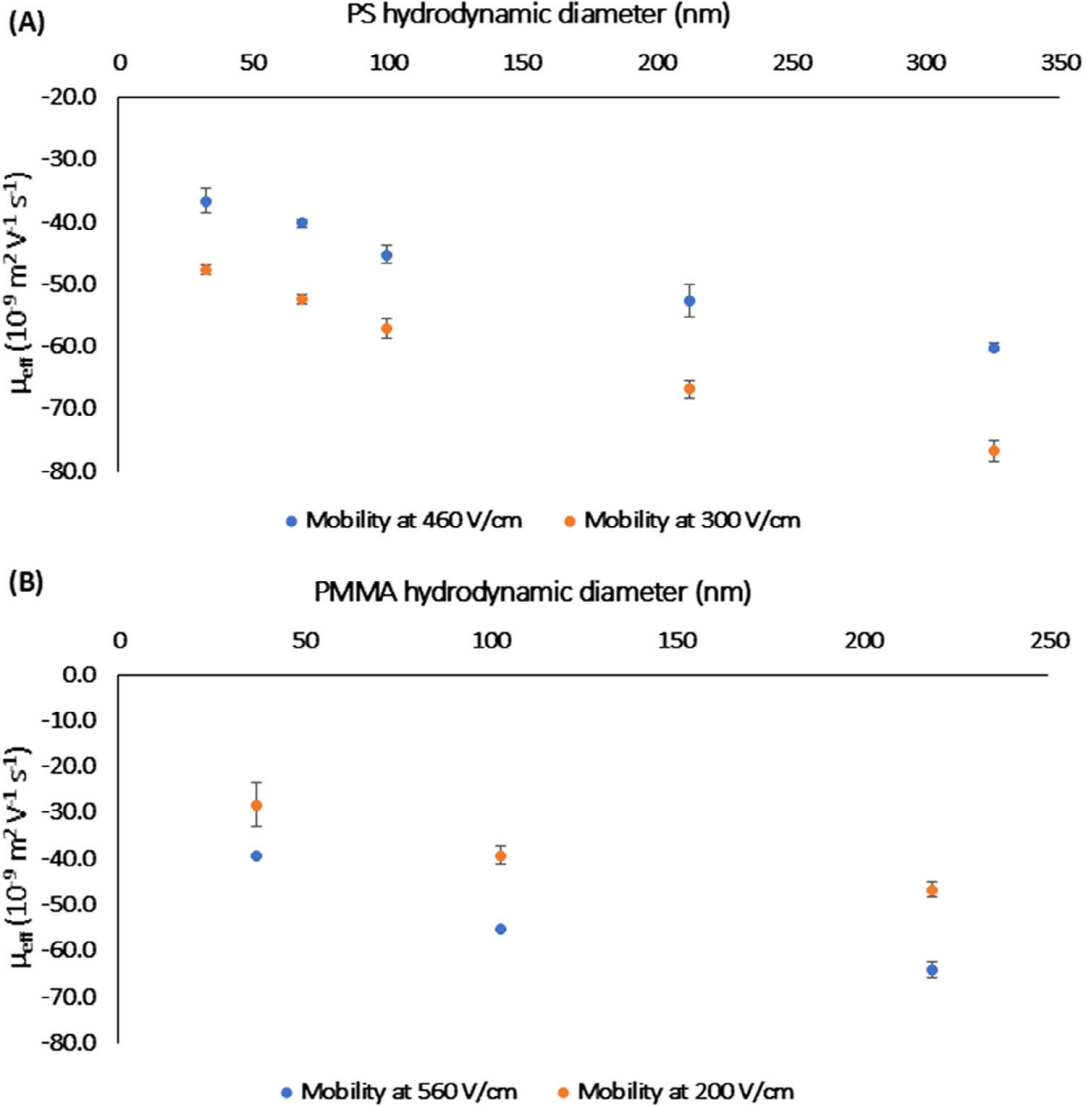
Measurements by CE of (A) electrophoretic mobility versus diameter of PS NPs at electric fields of 460 V cm^−1^ (electric current 57 µA) and 300 V cm^−1^ (electric current 30 µA); (B) electrophoretic mobility versus diameter of PMMA NPs at electric fields of 560 V cm^−1^ (electric current 40 µA) and 200 V cm^−1^ (electric current 18 µA).

For CE separation of PS NPs, electric field strength was 460 V cm^−1^, and corresponding mobility values are shown in Figure 2A from smaller to larger particle diameters. Even though the electric field strength was the same as in the above electropherograms, a shorter capillary length (50 cm) was employed for these measurements, resulting in an increase of absolute mobility values. Intensity of the electric current was 57 µA, with a gradual increase over time, as depicted in Figure S1A, and total input power was 1.71 W. This slight increase in electric current reflects a noticeable effect of Joule heating over time. Following previous reports [13, 14] in which temperature variation for BGE is assessed, temperature raise for CE separation of PS NPs under the aforementioned conditions might reach up to 5°C for a capillary with 50/375 µm id/od. Considering the total capillary length (50 cm) and an id of 100 µm (od constant), the actual BGE temperature could exceed 35°C, resulting in a temperature rise of 10°C. In this scenario, the cassette temperature may be set at 15°C to circumvent Joule heating effect and accurately measure electrophoretic mobility. Its values at a lower electric field strength (300 V cm^−1^), electric current being 30 µA, further confirmed this effect, as electrophoretic mobility increase was found more stable around 25°C, perhaps due to lesser Joule heating effect or reduced electric double layer (EDL) polarization effects. At higher electric fields, Joule heating may cause a localized temperature increase, reducing medium viscosity and enhancing Brownian motion to increase particle mobility [15, 16], even though effective mobility may decrease due to thermal effects on nanoparticle movement. It is also hypothesized that EDL polarization becomes more significant, further reducing the effective mobility due to ionic interactions between nanoparticles [17, 18], especially for smaller particles in the case of PS NPs. Experimental error bars shown in Figure 2A reflect a pronounced variability in measurements at higher electric fields, possibly because of increased sensitivity to temperature fluctuations.

For CE separation of PMMA NPs, electric field strength was 560 V cm^−1^, and mobility values followed the same trend as PS from smaller to larger particles (Figure 2B). Intensity of the electric current was stable at 40 µA, with a constant profile over time (Figure S1B). Following the normalization approach proposed in previous studies [13, 14, 19], the input power per unit length for the experimental conditions was calculated as 2.24 W m^−1^. Using the reference parameter of 0.33 W m^−1^ causing a 1°C temperature increase, the estimated rise in temperature for the presented setup is approximately 6.8°C above the nominal 25°C. This estimation suggests that the cassette temperature should be set at 18.2°C to prevent significant temperature fluctuations.

The stable electric current profile in Figure S1B demonstrates a reduced Joule heating effect, likely due to the narrower capillary id, enhancing heat dissipation as reported in previous findings [20, 21]. The observed behavior of PMMA NPs under varying electric field strengths aligns with theoretical predictions. The absolute mobility values increase at 560 V cm^−1^ due to reduced temperature effects and stable control of Joule heating [22, 23]. This behavior may be exhibited because of lower charge density for PMMA NPs, minimizing sensitivity to EDL polarization and localized thermal effects [24]. Viscosity drag prevails for larger particles, ensuring the expected electrophoretic behavior under elevated electric fields [25, 26]. Measurements at a lower electric field strength (200 V cm⁻¹), electric current being 18 µA, revealed a similar trend in the absolute values of electrophoretic mobility as PMMA NP diameter increases (Figure 2B), thus the effect of Joule heating cannot be neglected.

**FIGURE 3:**
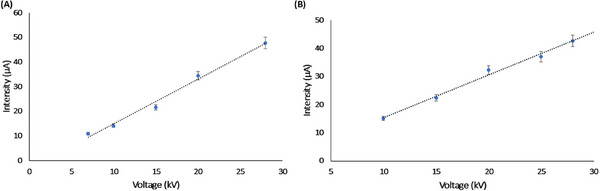
Current–voltage profile for CE measurements of (A) PS NPs in BGE containing 5 mM sodium phosphate dibasic with 5 mM sodium dodecyl sulfate at pH 8.9; (B) PMMA in BGE containing 2.14 M ammonium hydroxide at pH 11.9.

Figure 3 depicts the dependence of electric current on voltage for several values of separation voltage in CE. It is likely to infer that the trend in current is linear as voltage increases, even though it seems that Joule heating may be a variable out of control beyond 20 kV. Scenarios of high electric field strength values frequently take place in CE separation, therefore it is suggested to estimate temperature increase for the BGE in order to adjust cassette temperature accordingly.

In the case of LDV, voltage was set at 150 V, therefore electric field strength was 25 V cm^−1^, and intensity of the electric current generated was 2.2 mA (Figure S2A) and 3.1 mA (Figure S2B) when Zetasizer device was measuring mobility of PS NPs and PMMA NPs, respectively, estimated input power being 0.33 W and 0.47 W in each case.

For the PC cell employed in LDV (6 cm long, 4 mm id), temperature rise for mobility measurements will be mainly dependent on capillary id, as total length may not play a relevant role. The effect of Joule heating may be of a greater relevance in LDV compared with CE, even though it may be possible to estimate Joule heating by manual measurements of BGE temperature between LDV runs. The main drawback in the laboratory is the low sample volume (<1 mL) to fill in the PC cell, sample loss being also relevant and increasing difficulty in measuring temperature between runs. To avoid or circumvent the effect of Joule heating, it may be more practical to set applied voltage at a lower value than default (150 V), given the fixed length for PC cell.

Another limitation to be pointed out regarding conditions for PMMA NPs was the BGE employed, strongly alkaline (pH 11.9), therefore this scenario may not be beneficial for the PC cell, as this pH value is out of the recommended range for cell use. Disposing and replacing cell is indicated in this case. The Joule heating effect may also play a role in the increase of PMMA NP mobility values in CE, as the bare‐fused silica capillary was observed not to withstand after a certain number of injections (∼20) under these conditions, capillary replacement being often needed.

### 3.3 Evaluation of Potential Factors Affecting Mobility Measurements

From the aforementioned data, it can be hypothesized that capillary material and potential coating of the inner capillary wall may be a relevant factor for variability source between techniques. Bare fused silica capillaries without a coating have been employed in this work for CE measurements, whereas for LDV folded cells made of PC have been of use. Particles are prone to interacting with the capillary surface in CE owing to the high surface energy of the silica inner wall, possibly leading to undesired adsorption and measurement variability.

Several strategies, such as surface treatments or silica coatings, have been developed to circumvent this phenomenon [27], even for PS separation by particle diameter [28]. Nevertheless, the use of surfactants or additives in particle suspensions introduces additional challenges, as their adsorption or desorption onto capillary coatings may impact EOF mobility values in an unexpected manner.

To test the potential of a dynamic coating, experiments were conducted using Brij 35, a non‐ionic surfactant known for its stabilizing properties in CE separations [29, 30]. Brij 35 has been demonstrated to perform more efficiently for hydrophilic solutes than SDS and allow for compound separation that may not otherwise be possible when micelles are formed [31]. Furthermore, its non‐ionic nature may minimize electrostatic interactions with particle surfaces, ensuring separation stability. At 1% Brij 35 concentration, as shown in Figure S3, some sizes of PS NPs are aggregated and adsorbed on the capillary wall, demonstrated by spikes and tailing peaks present on the electropherogram, and separation is poor. At concentrations as high as 5% in the BGE, Brij 35 led to a reduction in aggregation degree of PS NPs, even though they cannot be separated. This occurrence is consistent with previous reports that excessive surfactant concentrations can induce micelle formation and interfere with analyte resolution [30, 31].

PC sheets for the LDV cell of use are generally not recommended for aqueous alkaline salts, ammonia gas and its solutions. These limitations in mobility measurements were not observed in CE, as universal capillaries constitute an advantageous feature. From an analytical point of view, it is of importance to consider reproducibility and bias, and perhaps a more resistant cell should be of use for LDV acquisitions to reduce variance. Nevertheless, the PC cell is most widely used in the laboratory for electrophoretic mobility measurements, also covering a scenario of budget wise decision‐making. The so‐called universal dip cell may be suitable to circumvent such limitations, even though the referred cell is 100 times costlier than PC cell, perhaps unaffordable in certain research groups.

**FIGURE 4:**
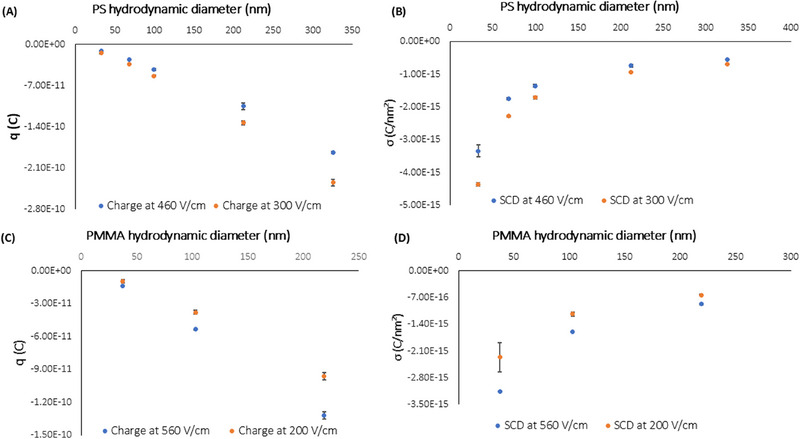
Graphs of (A) particle charge versus diameter of PS NPs at electric fields of 460 V cm^−1^ (electric current 57 µA) and 300 V cm^−1^ (electric current 30 µA); (B) surface‐charge density versus diameter for PS NPs at 460 V cm^−1^ and 300 V cm^−1^. Main conditions are as follows: bare fused silica capillary (50 cm length, 41.5 cm effective × 100 µm id x 363 µm od); cassette temperature 25°C; hydrodynamic injection during 5 s at 50 mbar; BGE 5 mM sodium phosphate dibasic with 5 mM sodium dodecyl sulfate at pH 8.9. (C) Particle charge versus diameter of PMMA NPs at electric fields of 560 V cm^−1^ (electric current 40 µA) and 200 V cm^−1^ (electric current 18 µA); (D) surface‐charge density versus diameter for PMMA NPs at 560 V cm^−1^ and 200 V cm^−1^. Main conditions are as follows: fused silica capillary (50 cm length, 41.5 cm effective × 75 µm id x 363 µm od); cassette temperature 25°C; hydrodynamic injection during 5 s at 50 mbar; BGE 2.14 M ammonium hydroxide at pH 11.9.

Another variable that plays a critical role is charge, and in turn SCD of particles, them both not necessarily scaling as size increases. Figure 4 shows the trend in charge and SCD for the PS and PMMA NPs assayed, making use of Equations (7) and (8) for corresponding calculations. As demonstrated with results herein shown, and also proven by our recent research [12], the trend in charge was found to be a negative straight line for PS NPs, and more importantly the negative SCD magnitude was exhibiting a decreasing trend as particle diameter increases, in a similar way to reported literature for spherical silica NPs [32].

The trend found in Figure 4A may be explained by the interaction between particle shape and EDL thickness, becoming comparable to particle radius for smaller particles and leading to partial charge screening [33]. As particle size decreases, the effect of surface charge becomes more pronounced due to the higher surface‐to‐volume ratio. A dramatic drop in SCD was observed for PS NPs below 100 nm, this decrease being slighter for NPs up to 300 nm (Figure 4B). For larger PS diameters, SCD magnitude reached a plateau and became almost independent of size, consistent with previous studies suggesting that contribution of surface charge diminishes as particle size increases [32, 34]. Regarding PMMA NPs, the trend in charge was also a negative straight line (Figure 4C), and a similar decreasing trend in the SCD magnitude was observed as particle size increased (Figure 4D). The sharp drop in SCD was occurring for the smaller particles, and kept slightly decreasing up to 200 nm, in an analogous manner to the behavior reported in spherical silica nanoparticles [32]. This trend in SCD may also explain the significant increase in absolute value of electrophoretic mobility for smaller particles, and the slight increase for larger particles, as EDL thickness may influence effective charge and particle mobility [35, 36].

It is also remarkable to highlight that capillary dimensions, particularly total length and id, are a potential factor impacting on *µ*_eff_ measurements. Narrow capillaries offer advantages such as enhanced heat dissipation owing to a higher surface‐to‐volume ratio and reduced particle‐wall interactions, contributing to consistent electrophoretic conditions at high electric field strengths. In this work, a balance was achieved by using standard capillary lengths (50–65 cm), which provided appropriate resolution for PS and PMMA NPs while maintaining analysis efficiency. For PS separation, a 65‐cm capillary ensured adequate resolution, while PMMA required only 50 cm, as longer lengths would result in unnecessary increments of migration times. These length values were selected considering physical constraints such as cassette dimensions and capillary wall durability. Commercial CE devices, capable of applying up to 30 kV, permit electric field strengths to range within hundreds of V cm^−1^, enabling users to optimize separation conditions. In this regard, the recommendation is beginning with low electric fields to assess separation feasibility and gradually increasing voltage if needed.

Capillary id also influences the electric current generated during a CE run. The equation *i* = *κ E* [37] is a variant of Ohm's law that relates the current density and the field strength at a specific point, wherein *i* is electric current density, *κ* is the specific electric conductivity of BGE, *E* is the electric field intensity, and *d* is capillary id. In this study, capillary id for CE was in the region of tens of micrometer, permitting electric current to be comprised in the µA range, whereas LDV exhibited currents in the mA range because of the wider id (mm region). Although electric current in the µA range may be accurately measured, low values in CE may amplify minor fluctuations in conductivity or separation conditions, potentially affecting *µ*_eff_ reproducibility.

An additional distinction between the two techniques lies in the particle size that can be analyzed. CE is better indicated for nanoparticles, as larger species may cause clogging in narrow capillaries. An upper size limit in the region of hundreds of nanometer is typical for CE, while PC cell for LDV may house even microparticles, possibly because of its wider id. This difference highlights the complementary roles of CE and LDV in particle analysis, the former offering simultaneous resolution of multiple particle sizes in a single run, whereas the latter requires separate measurements for individual particle sizes. The influence of EOF on *µ*_eff_ was minimal in this study, as CE values were corrected by subtracting EOF from apparent mobility. However, an electroneutral EOF marker namely dimethyl sulfoxide is indicated rather than solvent peak, the latter being employed in this study. The usage of a solvent as EOF marker may introduce variability, since its migration time need not be always identical with the migration time of the electroneutral compound [38]. Typically, EOF is not included in the *µ*_eff_ magnitude, as it is accounted for in CE through subtraction from apparent mobility, whereas LDV is capable of measuring the true electrophoretic mobility. That is certainly an alternative terminology to name *µ*_eff_, also known as net electrophoretic mobility. The region wherein this value is estimated is called stationary layer, as EOF is compensated by an opposite flow in the cell center. By means of LDV, it is also possible to gather simultaneously a value of electrokinetic (zeta) potential, directly related with *µ*_eff_ according to Equation (5) [39]. For the NPs herein reported, Henry function has been estimated to be equal to 1.5 according to Smoluchowski approximation for the scenario of an aqueous medium and moderate BGE concentration [40]. An increment in the absolute value of *ζ* will directly imply an increase in the absolute value of *µ*_eff_ for the particles subject to study. It is relevant to remark that *ζ* magnitude provides an indication of stability for particle dispersion, in such a way that a commonly accepted threshold of electrokinetic (zeta) potential value for stable aqueous suspensions is about ±30 mV [41]. Values below this figure may entail a constraint in determination of *µ*_eff_ for particles suspended in certain media.

It is also remarkable to point out a significant difference between CE and LDV with respect to number of items subject to analysis. In this regard, CE is able to resolve mixtures of different components, providing single peaks for every compound under selected conditions. Thus, it is feasible to derive *µ*_eff_ from individual migration times by means of velocity equations, obtaining several values of *µ*_eff_ with run replicates. By contrast, LDV only provides a sole value for mobility, regardless of the particle types present in the sample. As a result, separate LDV measurements needed to be carried out for every size of PS and PMMA NPs, involving individual preparations that increased laboratory work. Time analysis for both techniques is similar, as LDV is able to acquire three times a single sample in 5 min, which should be followed by a duplicate independent sample, together with manual pre‐ and post‐conditioning for PC cell. With regard to CE, an analysis for NP suspensions in this study lasted a maximum of 15 min, preceded by 5‐min capillary conditioning with BGE and followed by 5‐min capillary post‐conditioning with water. In principle, CE seems to be preferred as it can separate differently sized particles in a mixture during the same time than LDV employs for single sizes. In addition, both pre‐conditioning and post‐conditioning steps are automatically performed by the CE instrument with its inner pump, whereas this labor is carried out manually in LDV. Consequently, from a pragmatic point of view, CE approaches seem faster to determine mobilities for several species in a single analysis, while samples bearing individual sorts of particles are required for LDV in order to carry out separate measurements. In this perspective, LDV may be regarded as a complementary technique to verify whether trend in mobility figures aligns with CE experiences.

## 4 Concluding Remarks

Throughout this research, it has been demonstrated that both CE and LDV are reliable techniques for determining electrophoretic mobility of charged or charge‐induced particles. Joule heating is one of the most relevant factors playing a role in mobility measurements, therefore thermostating system needs to be properly guaranteed, or at least temperature variation is suggested to be carefully controlled. Nonetheless, overheating in LDV is more likely to occur than in CE under the experimental conditions assayed in this work. This limitation can be circumvented by acquisition of a cell withstanding stronger BGE conditions than PC cell. Separation voltage is also a potential variable when measuring *µ*_eff_ by both techniques, therefore the electric field strength can be regulated to minimize the effect of Joule heating. Additionally, a CE user will be in a position to shorten or lengthen capillary, and LDV analysts will be constrained to a specific capillary length when using the cell. In the scenario of a particle mixture, CE is clearly preferred because of its quickness and the feasibility to obtain simultaneous *µ*_eff_ values for several particle types in a single run. An assessment of trend in figures for *µ*_eff_ is also advisable by means of LDV mobility acquisitions. Although single particle types or diameters can be analyzed by LDV, its quick outcome and good reproducibility constitutes not only an efficient confirmation approach for CE figures, but an independent strategy for a whole study of this intrinsic parameter. Research groups aiming at studying separation mechanisms are suggested to employ mostly CE, and groups focusing on NP suspension stability are encouraged to opt for LDV, in both cases with no need for supplementary data provided by the other technique. It is believed that CE is more accurate to provide mobility values, mainly because it is feasible to alter separation voltage, capillary length, and id, or injected volume, thus enabling to compare data between different laboratory experiences. Concerning LDV, among the critical variables only voltage may be amended, and the rest of conditions are given by the cell of choice, measurements being also performed at fixed values of back‐scattering angle, thus single figures of mobility are provided without further tests. In the end, CE and LDV may be used either separately or jointly, although it is recommended to count on both instruments to gather a deeper insight into electrophoretic mobility of NPs, a unique parameter only achievable by such techniques.

## Conflicts of Interest

The authors declare no conflicts of interest.

## Supporting information



Supporting Information

## Data Availability

The data that support the findings of this study are available from the corresponding author upon reasonable request.
