# Supplementary material for: Assessment of Electrophoretic Mobility Determination in Nanoparticle Analysis: Two Parallel Techniques Converging in a Distinctive Parameter
Source: Electrophoresis. 2025 Apr 29;46(11-12):743–51. doi: 10.1002/elps.202400132 (PMC12366277; doi:10.1002/elps.202400132)
Supplement: Supplementary file 1 — Supporting Information [file ELPS-46--s001.docx]

**Supporting Information**

**Assessment of electrophoretic mobility determination in nanoparticle analysis: two parallel techniques converging in a distinctive parameter**

Carlos Adelantado^1,*^, Jan Jordens^1^, Stefan Voorspoels^1^, Milica Velimirovic^1^, and Kristof Tirez^1^

^1^Flemish Institute for Technological Research (VITO), Boeretang 200, 2400 Mol, Belgium

*Corresponding author: Carlos Adelantado – Materials and Chemistry Unit, Flemish Institute for Technological Research (VITO), Boeretang 200, 2400 Mol (Belgium); (orcid.org/0000-0003-1360-3110); Email: [carlosadelantado@gmail.com](mailto:carlosadelantado@gmail.com)

**
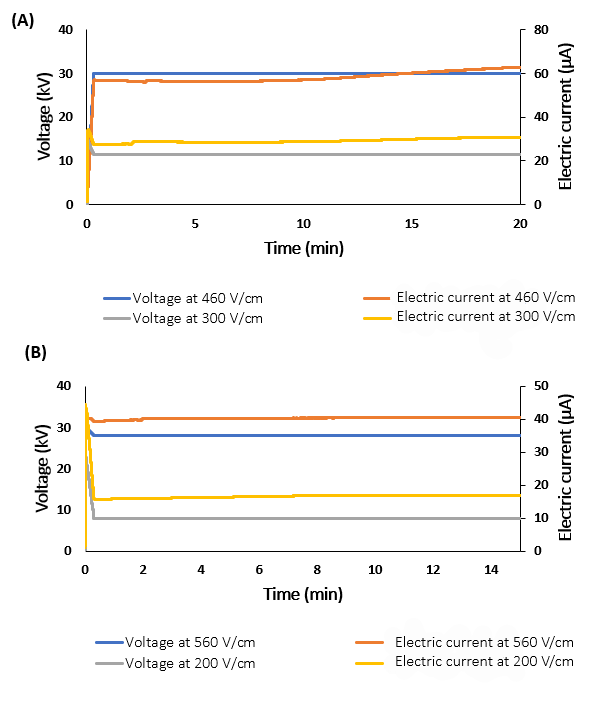
**

Figure S1. Measurements by CE of: (A) current-voltage profile for PS NPs at 460 V/cm and 300 V/cm. Main conditions: bare fused silica capillary (50-cm length, 41.5 cm effective × 100-μm id); cassette temperature 25ºC; hydrodynamic injection during 5 s at 50 mbar; BGE 5 mM sodium phosphate dibasic with 5 mM sodium dodecyl sulfate at pH=8.9; (B) current-voltage profile for PMMA NPs at 560 V/cm and 200 V/cm. Main conditions: fused silica capillary (50-cm length, 41.5 cm effective × 75-μm id x 363-µm od); cassette temperature 25ºC; hydrodynamic injection during 5 s at 50 mbar; BGE 2.14 M ammonium hydroxide at pH=11.9.


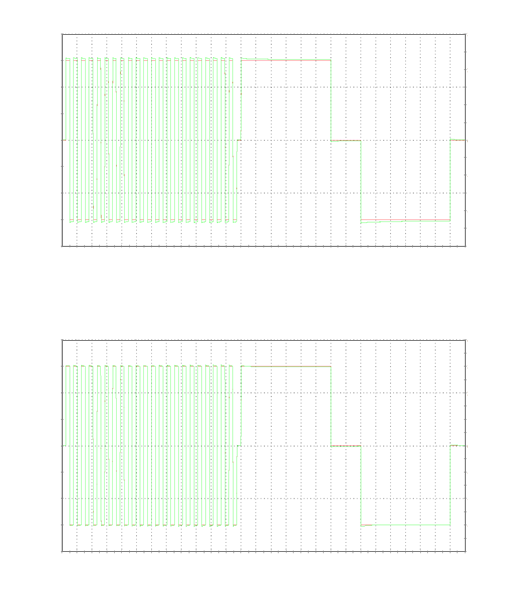


(A)

(B)

Voltage (V)

Voltage (V)

Current (mA)

Current (mA)

Time (s)

Time (s)

**Zeta Potential Voltage and Current**

**Zeta Potential Voltage and Current**

200

100

-100

0

3

2

1

0

-1

-2

4

3

2

0

-1

-2

1

-3

200

100

-100

0

0

0.5

1.0

1.5

2.0

2.5

0

0.5

1.0

1.5

2.0

2.5

Voltage

Current

Voltage

Current

Figure S2. Current-voltage profile by LDV for measurement of electrophoretic mobility of: (A) 31-nm PS NPs in a BGE containing 5 mM sodium phosphate dibasic and 5 mM sodium dodecyl sulfate (pH=8.9); (B) 38-nm PMMA NPs a BGE containing 2.14 M NH_4_OH (pH=11.9). Main conditions: laser module of He-Ne; power input 4 mW; laser wavelength 633 nm; cell temperature 25 °C; back-scattering angle 173°; electric field strength 25 V/cm.


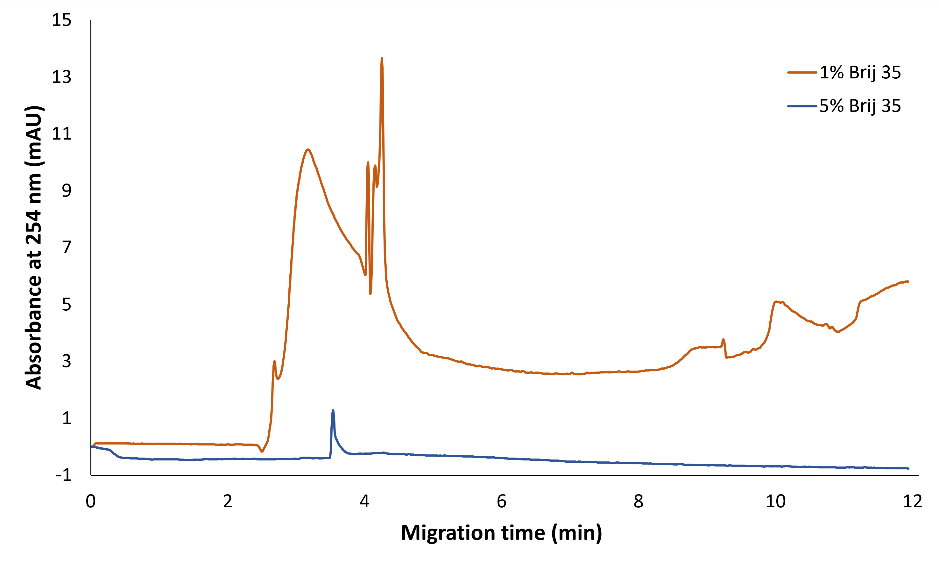


**Figure S3**. CE electropherograms of PS NPs. Analytes present: 31 nm PS 6.74x10^12^ particles mL^-1^, 62 nm PS 8.43x10^11^ particles mL^-1^, 92 nm PS 2.50x10^11^ particles mL^-1^, 202 nm PS 2.27x10^10^ particles mL^-1^, 303 nm PS 6.74x10^09^ particles mL^-1^. Main conditions: bare fused silica capillary (65-cm length, 56.5 cm effective × 100-μm id x 363-µm od) dynamically coated with Brij 35 (1-5%); cassette temperature 25ºC; hydrodynamic injection during 5 s at 50 mbar; separation voltage 30 kV; BGE 5 mM sodium phosphate dibasic with 5 mM sodium dodecyl sulfate.
